# Quantifying Dwell Time With Location-based Augmented Reality: Dynamic AOI Analysis on Mobile Eye Tracking Data With Vision Transformer

**DOI:** 10.16910/jemr.17.3.3

**Published:** 2024-04-29

**Authors:** Julien Mercier, Olivier Ertz, Erwan Bocher

**Affiliations:** MEI, School of Engineering and Management Vaud, HES-SO, Switzerland; Lab-STICC, UMR 6285, CNRS, Université Bretagne Sud, Vannes, France

**Keywords:** Mobile Eye Tracking Methodology, Dynamic Area of Interest, Dwell Time, Frame-byframe analysis, Vision Transformer, Location-based Augmented Reality, Educational Technology

## Abstract

Mobile eye tracking captures egocentric vision and is well-suited for naturalistic studies. However, its data is noisy, especially when acquired outdoor with multiple participants over several sessions. Area of interest analysis on moving targets is difficult because A) camera and objects move nonlinearly and may disappear/reappear from the scene; and B) off-the-shelf analysis tools are limited to linearly moving objects. As a result, researchers resort to time-consuming manual annotation, which limits the use of mobile eye tracking in naturalistic studies. We introduce a method based on a fine-tuned Vision Transformer (ViT) model for classifying frames with overlaying gaze markers. After fine-tuning a model on a manually labelled training set made of 1.98% (=7845 frames) of our entire data for three epochs, our model reached 99.34% accuracy as evaluated on hold-out data. We used the method to quantify participants’ dwell time on a tablet during the outdoor user test of a mobile augmented reality application for biodiversity education. We discuss the benefits and limitations of our approach and its potential to be applied to other contexts.

## Introduction

**Figure 1.**
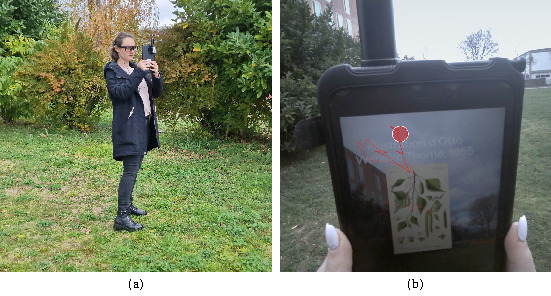
(a) A participant wearing a head-mounted mobile eye tracking device (Tobii Pro Glasses 3). (b) The front camera captures egocentric vision. The overlaying red dot representing gaze is added during post processing with the manufacturer’s software.

Eye tracking enables the measuring of various features related to an individual’s eye movements, providing a glimpse into their cognition and behavior (8). It is an unobtrusive, objective measuring tool for quantitative data that can be applied to various diagnostic analytics in fields like usability, psychology and neurology research, but also clinical rehabilitation, treatment, training, or even used as a natural interface (11; 22). Usually, eye tracking data act as a dependent variable and are linked to an independent variable for further interpretation: Studies often consist in observing the impact of external, semantic stimuli (e.g. looking at something, operating an interface, etc.) on a specific metric (e.g. dwell time, gaze position, pupil size, fixation sequence, etc.) (8). Despite being used in a growing range of contexts, many eye tracking measures are little known (31) and new methods to extract knowledge from eye tracking data are constantly emerging (44), which reflects the technique’s relative lack of maturity (30). Eye tracking data is often of uneven quality, making it notoriously difficult to analyze (2). Stationary eye trackers’ use is limited to controlled environments, and usually only operates with screen interaction. On the other hand, thanks to head-mounted devices (see Figure 1(a)), mobile eye tracking (MET) enables the recording of egocentric vision “in the wild” while engaging in everyday tasks (30), enabling naturalistic studies. Apart from the egocentric camera, inward-facing sensors typically operate at speeds from 25 Hz to 250 Hz (3). These sensors’ data is used to calculate the direction of the gaze and an overlaid visual marker may be marked on top of the egocentric video, as seen in Figure 1(b). MET offers an unprecedented opportunity to look through someone else’s eyes and momentarily step into their shoes. In usability research, MET can be used to identify key issues such as ill-informed use or challenges in spatial navigation. MET offers an optimal freedom of movement, enabling more realistic interactive experimental settings (14). This freedom comes at a price: precision, accuracy and sample rate are all decreased, resulting in data of even more erratic quality than its stationary counterpart (2; 5; 8; 30; 37). When MET is used outdoors, output data is even more challenging (12). When data collection takes place over multiple sessions, spanning several weeks, and covering large areas, as is common in naturalistic studies, the fluctuating environmental conditions further hinder data homogeneity.

Naturalistic studies featuring MET often rely on the use of areas of interest (AOIs). AOIs are regions in the visual field that hold semantic significance in the context of a given study. AOI dwell time (19) is the sum of the durations of fixations within the AOI. This metric can be interpreted as the total amount of time a participant gazed at the AOI. Dwell time may be subjected to statistical analysis to examine differences between conditions or AOIs, or inference about the cognitive processes it reveals, based on additional background theory the study relies upon (30). Although AOI-based methods represent a tool of choice for MET data analysis, they are often limited by variability in shape, size, and general definition of AOIs. Researchers often don’t include their definitions of AOI as part of their method (40). This variability decreases the reproducibility of research and makes inter-study comparison difficult (41).

AOIs pose specific challenges when used in combination with outdoor data (18). MET devices manufacturers advertise features such as “automated” and “dynamic” AOI that presumably allow the mapping and tracking of moving AOIs. But based on the examples show on tutorial videos, these refer to either a moving target, viewed from a static point of view or a static target, viewed from a moving point of view. In these situations, either the point of view or the AOI does not move, and the trajectory of the AOI remains much more linear. The light conditions under which objects are captured tend to vary less, making them easier landmarks to map and track by computer vision algorithms. In most MET naturalistic studies however, both the egocentric camera and the tracked object are moving independently, causing the latter to undergo abrupt, nonlinear changes (trajectory, size, shape), be subjected to contrasting light conditions, and even disappear and reappear (28), which makes traditional computer vision tracking algorithms ineffective. Admittedly, manufacturers’ tools are primarily devoted to the analysis of objects that move in a linear way and at a constant speed, which limits their usefulness to the analysis of data captured under laboratory conditions. These tools are usually unable to operate on noisy field data (2; 18). Because continuous data of reasonable quality are key for tracking such objects, performing data analysis involving moving AOIs is difficult. As pointed out by several researchers (5; 15; 23; 26; 28; 30), there is a gap in available, accurate, reliable, structured, easy to use, and automated methods to analyze eye tracking data in combination with moving objects. Moving AOIs analysis usually must be carried using bespoke computer vision methods. As a consequence to the unavailability of solutions, MET data is still often processed manually (24; 39; 41), which is very time-consuming and has major implications for the widespread dissemination of this methodology (23; 41).

In this paper, we present a method for post hoc automatic detection of users’ focus on moving AOIs by using one of the most up-to-date deep learning architectures for computer vision (see Appendix A). We evaluate the accuracy of the proposed method and discuss its potential for knowledge extraction from MET data beyond our own use case. We describe a comprehensive and repeatable pipeline ranging from data preprocessing, model training, hold-out validation, inference, post-processing, and data visualization, aiming to make the method repurposable to other researchers with comparable needs. We present our use case: MET data collected during the usability test of a location-based augmented reality application for biodiversity education. One of the goal of this user test was to measure participants’ dwell time on the tablet screen as opposed to looking up at the natural environment during 15-minute sessions (35).

## Background

### Mobile Eye Tracking for Educational Technology Research

The rapid development and adoption of mobile technologies has sparked numerous research projects aimed at improving learning processes using information technologies. While mobile screen can be beneficial when used for education, it has drastically increased since the COVID pandemic (33) and an excessive or harmful use by young audiences is a societal concern. Considerations of the content and context as well as considerations on the design of the interfaces that shape educational technology are essential for guidelines that are practical and operationally relevant (25). While excessive mobile screen time and its impact highly depend on the context, technology was found to dominate user experience in a problematic way in 70% of the examined mobile learning projects (17). Methods used to quantify screen time vary from one study to the next and often lack precision, making it difficult to make informed decisions (25) or even compare results. It is therefore important and useful to monitor and track screen interaction dwell time in mobile learning experiments with methods that are scientifically based, aimed at objectivity and reproducibility. In a typical outdoor user study that includes eye tracking, participant-generated data is recorded during multiple sessions, over extensive areas, and under varying lighting and environmental conditions (12; 28). This process can quickly result in large amounts of noisy data that is difficult to process. As a result, extracting any kind of actionable knowledge from this type of data or that of others that present similar challenges is difficult. In our use-case, MET data was gathered during a mobile application for biodiversity education’s user test (35). We wanted to measure the ratio of dwell time participants interacted with the mobile device screen to gauge the role played by technology in the use of our system.

### Deep Learning for Eye Tracking Data Analysis

**Figure 2.**
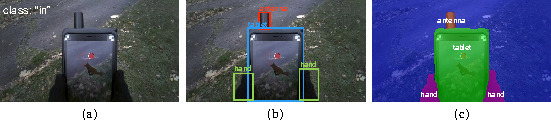
(a) Image classification: an entire image is assigned with a label, or “class”. Our approach consists in training a model to detect whether the red gaze marker is located within the tablet. (b) Object detection: several objects are detected as well as their locations as rectangular frames. While more insightful than image classification, determining if the red gaze marker lies in the tablet would require an extra step of geometric calculation to cross-reference the marker’s coordinates with that of the tablet’s area, and rectangular shape of the latter makes it prone to inaccuracies. (c) Semantic segmentation: several objects are detected, and the detail of their contour. Using this approach to compute dwell time on the tablet would also require extra geometric calculation, without being prone to inaccuracies, because the contours accurately fit the detected objects.

The benefits of using computer vision algorithms powered by deep learning for MET analysis has been repeatedly demonstrated and their use have increased over the last few years. Three common methods stand out in this context: image classification, object detection, and semantic segmentation (42). With image classification, input images get labeled from a set of predefined classes, as illustrated in Figure 2(a). With object detection, multiple objects are labeled on input images and localized by drawing a rectangular bounding box around them, as illustrated in Figure 2(b). With semantic segmentation, objects are labeled and accurately contoured, and may include surfaces (sky, ground, water…), as illustrated in Figure 2(c). These methods (see Appendix A) are especially relevant for analysis that include nonlinearly moving AOIs, which are very difficult to track with deterministic computer vision algorithms.

A few works addressed the problem of mapping gaze to moving objects or AOIs in MET data. Deep learning models are applied in a variety of ways, but in most cases researchers used a convolutional neural network (CNN, see Appendix B) object detection model to draw boundaries around the objects, and then cross-referenced these with gaze data (5; 7; 26; 43; 46; 47; 49; 50). Kumari et al. (26) compared the performance of three CNN object detection models to analyze MET data. Venuprasad et al. (49) used object detection with clustering and further cross-referenced the gaze point coordinates. Tzamaras et al. (47) used an object detection model in combination with an additional, custom-trained object detection model to track the plotted red gaze marker. Sümer et al. (46) used a face detection model and cross-referenced the raw gaze points coordinates to examine a teacher’s attention to their students. Callemein et al. (7) present a system that detects when gaze focuses on other persons’ face or hands. Wolf et al. (50) mapped gaze (fixations) to moving AOIs with Mask R-CNN, with the advantage of operating in real time. Barz and Sonntag (5) also presented real-time object detection using pretrained models without fine-tuning and introduce an evaluation framework for automatic gaze to AOI mapping. Silva Machado et al. (43) matched the detected bounding boxes with participants’ fixations using a sliding-window approach with a MobileNet CNN model. Rather than using the model for inference on large data for automatic analysis, several tools are multitask and offer interactive visualization for manual annotation (4; 27; 28, 29; 38). Kurzhals et al. (28) introduced an interactive labeling tool with automatic clustering combined with an analysis system. They went on to develop image-based (29) and gaze patches techniques (27) for dynamic AOI annotation that are conceptually similar to our proposed idea of merging gaze data with video. Barz et al. (4) have implemented an approach based both on image classification and object detection. They used a few-shot learning method for its adaptability, with a 50-layer CNN (ResNet50). The classifier makes suggestions to the human operator of an interactive annotation tool. Based on user feedback, the model can be re-trained for better performance. Panetta et al. (38) used semantic segmentation to create a graphical user interface that enables dynamic MET data visualization. Finally, some researchers have introduced methods to analyze egocentric videos without gaze data. Ma et al. (32) use an optical flow algorithm, object and hand segmentation on egocentric videos to analyze the participant’s activity. Bertasius et al. (6) determine the object of attention by analyzing action in egocentric videos.

While approaches based on object detection or semantic segmentation offer many benefits, the models only play a role in first step of AOI analysis by mapping and tracking the detected object dynamically over time. After this has been done, the AOIs geometries discovered by the models must still be cross-referenced with the gaze coordinates using traditional geometry formulas. Object detection defines rectangular bounding boxes around detected objects that do not wrap them closely, which may bias the analysis (18; 26; 41). The gaze point may be located inside the bounding box but on an empty area, thus returning a false positive or even targeting another unwanted object in the background. Several boxes may also overlap. Semantic segmentation addresses this issue, since AOIs are closely shaped after the objects (39). However, it is more time-consuming to prepare training data for object detection or semantic segmentation than for image classification. Because these models’ tasks include image classification in addition to other tasks (localization, detection, or segmentation), these models would perform with less accuracy for the analysis at hand than image classification-only models: SOTA for object detection on the COCO benchmark is 66% [BOXMAP metric] (53), 53.4% for semantic segmentation [mIoU metric] (13), and 93.4% for multi-label image classification [MAP metric] (52). Overall, object detection and semantic segmentation seem needlessly sophisticated for the type of AOI analysis at hand: it may not be necessary to know the location of an AOI to determine whether the gaze point is on it.

## Methods

### General Approach

The question we sought to answer was: What percentage of the experiment’s time do participants spend dwelling at the screen? Although this analysis seems conventional, the means of carrying it out are not obvious, as we found out. At the time of data collection, we expected that the analysis software provided with the devices may allow us to perform the AOI dwell time analysis and thus answer our question. In the processed egocentric videos, the overlaying red gaze marker is seen entering and exiting the area of the 8-inch tablet handled by participants during the outdoor test. However, the tablet is viewed from constantly changing angles and often disappears from the camera’s field of view. As a result, any attempt to track its contours using the manufacturer’s moving AOI tool failed immediately. Subsequently, we tested two deterministic computer vision algorithms (Lucas-Kanade optical flow and template matching with normalized correlation coefficient), but they quickly proved inoperant: With optical flow, the process got interrupted as soon as the tracked object (the tablet) was not visible in a frame, while the smallest variation in the data (gaze point size or position) caused the template matching methods to dysfunction. Consequently, we figured that a classification model could detect the presence of the tablet in static frames extracted from videos. We also assumed that the same model could detect whether an overlaid gaze marker is located within the tablet or outside of it, provided the model is trained on labeled data representing the features of each category (in/out). Unlike other researchers who sought to address this challenge, we did not consider the use of an object detection model, despite the suitability of this approach, which mimics the steps of a manual analysis. In comparison with previous research, we introduce a more cunning, blind, and minimalistic approach featuring the use of SOTA ViT architecture (see Appendix C) for image classification where each frame needs not be parsed as thoroughly. Instead, the model learns to classify frames of the egocentric videos based on their visual appearance, including the visual marker at gaze position. To our knowledge, such a minimalistic method was not described or documented elsewhere, possibly because it might not meet typical additional analysis requirements of other use cases. In our situation, this seemed to be the most suitable tool and it occurred to us that the method could offer a straightforward solution in some other circumstances, too. Our classifier naturally learns whether the overlaying gaze marker is located upon a specified object or not. It can be used to identify if gaze points are directed to as many objects as are found in the data, without any need for additional geometric calculations. As discussed in the introduction, although their task is different, classifiers’ SOTA is higher than object detection or semantic segmentation models. In the following sections, we outline the steps we took to carry out AOI dwell time analysis on our mobile eye tracking data.

### Data Collection

Data was collected from November to December 2022 during the user test of a location-based augmented reality application (35). We used Tobii Glasses 3 (see Figure 1(a)), which are lightweight but do not play well with prescription glasses wearers. Out of 54 participants in total, 48 were able and agreed to wear an eye tracking device. 7 recordings failed and were not saved on the SD card, resulting in a final sample of 41 participants’ egocentric videos captured at a sample rate of 50 Hz. Lasting approximately 15 minutes each, the total data amounted to 11 hours of video to analyze. The captured data was downloaded on a computer and processed with the Tobii Pro Glasses Analyzer software. In the software, we combined the egocentric videos with the unfiltered (as opposed to the optional “fixation”, “noise” or “low pass” filters available) gaze data, which is standard procedure for eye tracking data visualization. The resulting videos feature an overlaying red marker at gaze location were exported (see Figure 1(b)).

### Data Pre-processing

We extracted frames from our 11 hours of video data at a rate of 10 frames per second, for a resulting 11 × 60 × 60 × 10 = 396000 images of 1920 × 1080 pixels. These figures are the result of a balance between a sampling rate that allows the rendition of smooth videos and a total amount of data points that can be easily inferred by the trained model on a conventional computer during later stages. The frames were organized into 41 directories (one per participant) for post-processing and data interpretation. The frames were rescaled to a square aspect ratio–as is the norm for computer vision models–with a resolution of 320 × 320 pixels. Although the pretrained model we used was trained on input data of 224 × 224 pixels, it is common practice and beneficial for accuracy to use a slightly higher resolution for best results with fine-tuning (21).

### Training Set and Manual Image Labeling

**Figure 3.**
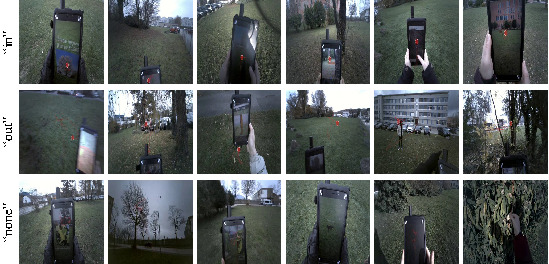
Samples of the resized and labeled dataset. It comprises three classes: “in” where gaze point is in the tablet screen; “out” where gaze point is located outside of the screen; “none” where gaze point was not resolved and there is no overlaid visual marker.

In preparation of training, we randomly selected 1.98% of our dataset (= 7845 images) proportionally to each of the participants’ samples to prevent over/underfitting. The size of the training set was defined based on the estimated time it would take to manually label. In our situation, the tablet represents the moving AOI. We want the model to detect whether the gaze marker is in or out, so we defined two categories, based on the possible locations of the marker: “in” or “out”. We realized some of the frames in our dataset did not feature any gaze marker. This is due to the naturalistic setting of the data collection: sometimes the inward-facing cameras can’t retrieve the necessary data to resolve and estimate the gaze direction. We therefore added a “none” category for these frames without markers (see Figure 3). We used the free and open source tkteach tool (36) to label the frames. The process took 2 hours and 24 minutes. Measuring time will be useful as a reference to calculate the time saved by using our automated approach during later stages. The selected samples were distributed across the three categories as follows: “in” = 4803 (61.22%); “out” = 1795 (22.88%); “none” 1247 (15.9%).

### Model Training

We used Huggingface’s “transformers” library, an API for loading checkpoints and datasets, fine-tuning, and deploying models. The process of fine-tuning a ViT model involves adapting a pretrained, general-purpose model to a smaller downstream task, such as image classification into the “in”, “out”, and “none” classes of our dataset. This enables the transfer of the pretrained model’s ability to represent images and extract their most meaningful features. Technically, fine-tuning consists in replacing the model’s prediction head (e.g., the last one or two layers of the neural network) and creating a new linear (feedforward) layer with our number of classes to perform a new classification task. The transformers library has a “ViTModel” class that will load a bare ViT Model that only outputs raw hidden states, without any specific head on top. We chose a ViT-Base 16 checkpoint (16), which is a transformer encoder model pretrained on the large ImageNet-21k dataset (9) which contains 14 million images cropped at 224 × 224 pixels and 21843 classes. It was fine-tuned on the mid-sized ImageNet 2012 dataset (42) which contains 1.3 million images for 1000 classes (10). We chose the “base” version which is reported to perform well on small datasets whereas ViT-Large and ViT-Huge–which contain more hidden layers of bigger sizes–will underperform when trained on small datasets. ViT-Base 16 reports 88.55% accuracy on ImageNet classification, while SOTA’s current top-1 for ImageNet is 92.4% (45). To train the ViT model with our data, we apply specific transformations to images so that the data fits the model’s expected input format. The pretrained model comes with a configuration file that specifies the appropriate size, image mean, and standard deviation for the architecture we use. A specific *transformers* class processes our data points and turns them into multi-dimensional matrices (torch tensors) which in this case are 3D arrays: two dimensions of size 224 for each line/column in the image and one dimension of size 3 for the RGB value of each pixel. We then load the formatted dataset and the parameters it comes with, such as the number of classes, corresponding to the number of different labels in the training set. This creates a classification head with our own classes by defining the number of neurons in the last classification layer. At this point, training a custom model typically includes a data augmentation step: transformations (such as zoom, crop, inversion) are applied on the training set to artificially expand it and bring diversity, to foster the model’s ability to generalize. However, if future input data is finite, already known and well-represented in the training data, the model won’t need to generalize much, and data augmentation is therefore not useful. Finally, a series of hyperparameters is set to train the model, including but not limited to:

- Epochs: the number of times the model should process the entire dataset. We first ran a test with 3 epochs then further trained the model for 10 epochs, with the instruction to save checkpoints files at the end of each epoch.
- Batch size: the number of images that are processed per step. The bigger the value the faster the training, but it is generally limited by the available RAM and its ability to process large amounts of data simultaneously. We used a batch size of 24, which is standard.
- Loss function: the type of algorithm used to minimize the loss value. Cross-entropy loss was used. Its output, ranging from 0 to 1, represents how far off the model’s predictions are from ground truth. The smaller the loss, the best the model performs (both on training and unseen data) with the current weights. Together with accuracy, it may be used to determine the optimal checkpoint.
- Learning rate: the speed at which adjustments will be made in the weights of our neurons relative to the loss gradient descent. If the learning rate is too fast, the optimal weights will be overlooked, and we will miss the optimal checkpoint. If it’s too small, it will take too many steps to attain the optimal solution. An optimal rate should see the loss progress rather than jump up and down. We kept the pretrained model’s preset value of 5 × 10^−5^.
- Evaluation metric: a model’s performance may be evaluated upon its upon accuracy, precision, or recall, during and after training. We used accuracy, which represents how often the model is correct overall.

After (i) the training set was loaded; (ii) the headless pretrained model was loaded; and (iii) the hyperparameters were set, we started a training session set for a duration of 3 epochs by connecting to a NVIDIA T4 GPU through Google Colaboratory–a hosted Jupyter Notebook service providing free and paid access to computing resources. The training was set to evaluate the checkpoint at the end of each epoch, by testing its accuracy on a split of the dataset that was not used for training. The session lasted 11 minutes 47 seconds and the evaluation metrics reported 99.11% accuracy on the test data (see Appendix D). Observing that this peak accuracy was reached upon epoch no. 3, we resumed training on the last checkpoint with the same parameters for 10 additional epochs, to see if performance could be maxed. This additional training lasted 45 minutes and 48 seconds, but the training and validation loss curves and the accuracy plateaued after epoch no. 6 (see Appendix E). The training was set to save each session’s most accurate checkpoint, which were the one evaluated at epoch no. 3 and no. 6 (both underlined in Appendix D).

### Hold-out Validation

Because fine-tuned models are highly customized, they cannot be benchmarked against one another, and their performance can mostly be appreciated contextually. Fine-tuned models are gradually easier to train efficiently as their task simplifies (i.e., from Imagenet’s 1000 categories to our 3). In machine learning, data scientists often train models on large training sets and test them on smaller amount of data. This is due to widespread belief that the larger the training set, the better the model will perform. However, the actual goal of a model is to be efficient when inferring on unseen data. Therefore, the training set shouldn’t be larger than required to meet this purpose and it may be useful instead to reserve a fair part of the labeled data for test purposes. If a model performs well on unseen data, it will likely perform well in a production environment. This is why hold-out validation of a model is made on an unseen dataset. Considering that our model was aimed at analyzing a finite quantity of specific, noisy data, its accuracy should be evaluated internally rather than based on its ability to generalize on external datasets. Additionally, rather than relying exclusively on the accuracy metrics as measured after each epoch as part of the training pipeline on the test set (automatically split from the training set), it is good practice to submit the model to realistic testing conditions, with a hold-out dataset that is consistent with the model future working conditions for additional evaluation. To that end, we used the frames extracted from one of the 41 participants’ video as a new hold-out test set. The goal is to perform hold-out validation of both the accuracy of our models and that of the manual labelling, for comparison. The video is 17 minutes and 20 seconds long, which represents 10402 frames that we labeled manually. From those, we removed 192 frames that were also part of the training set to avoid data leakage. The manual labeling took 3 hours and 43 minutes, which gives us an additional reference to calculate the time saved by using our automated approach on the rest of the data. We then ran the same data in parallel through both saved checkpoints and logged the classification results. It took 55 minutes to infer the 10210 frames through one model. Given the large number of frames, it’s just as likely that errors were made during manual labeling as by the classification models themselves. We therefore compared the results of each method (manual, model v1, model v2) and singled out frames for which at least one of the three methods had diverged. In 97.76% (9981/10210) of the cases, all three methods returned the same label, indicating a high probability of correctness. We manually reviewed the remaining 2.24% (229/10210) of divergent results and reviewed them individually to establish ground truth. Upon reviewing, it turns out the manual labelling was wrong in 86 out of the 229 contentious frames, which represents an overall accuracy rate of 99.16% on the entire dataset. Model v2 (checkpoint saved after epoch no. 6) was wrong in 126 out of the 229 contentious frames, which represents an overall accuracy rate of 98.76% on the entire dataset, which is 0.22% below the “advertised” 98.98% accuracy calculated during training. Finally, Model v1 (checkpoint saved after epoch no. 3) was wrong in only 67 out of the 229 contentious frames, which represents an overall accuracy rate of 99.34% on the entire dataset, which is 0.23% higher than the expected 99.11% accuracy reported during training. This could be explained by the fact that the manually labelled data used for training was less accurately labelled than the automatically labelled data against ground truth. See Table 1 for a summary of the hold-out validation results.

**Table 1.** Hold-out validation results.

**Method**	**“in”**	**“out”**	**“none”**	**Errors^1^**	**Accuracy^1^**
Ground truth^2^	5714 (55.96%)	4298 (42.1%)	198 (1.94%)	ø	100%
Model v1	5691 (55.74%)	4318 (42.3%)	201 (1.96%)	67 (0.66%)	99.34%
Manual labeling	5726 (56.08%)	4327 (42.38%)	157 (1.54%)	86 (0.84%)	99.16%
Model v2	5608 (54.93%)	4394 (43.07%)	208 (2.04%)	126 (1.24%)	98.76%

^1^ Error rate was calculated relative to ground truth, which we subtracted from 100% to obtain accuracy rate. ^2^ Ground truth was established by manually assigning a new label to each frame for which Model v1, Model v2 and manual labelling returned different results.

**Figure 4.**
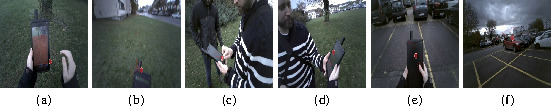
Samples of contentious frames: (a) & (b) Both models tended to mislabel the marker on the tablet’s edge, where manual labelling is also difficult. (c) & (d) Model v2 mislabeled other humans holding the tablet. (e) Concrete-only backgrounds were mislabeled more often than vegetation-backgrounds. (f) A red car in the back, no model detected the (also red) gaze marker.

Additionally, in 95 out of the 229 contentious frames, both models returned different results, hinting at their individual biases. Upon looking closely at the frames on which the models differed, most situations saw the gaze point located on the very edge of the tablet (see Figure 4(a) & 4(b), where it was hard to draw the line even during manual labeling. Model v1 tends to consider these debatable situations as “in” whereas Model v2 often ruled them “out”, demonstrating an interesting discrepancy in their individual sensibilities. Model v2 also seemed to make more mistakes when another human was holding the tablet (see Figure 4(c) & 4(d))–which admittedly changes the perspective and visual appearance of the tablet–indicating that the model was possibly overfitting. Most situations where both models were wrong included frames where the gaze point was on the edge of the tablet as well, but also seldom cases, such as when the background contained no grass or urban elements such as cars or concrete (see Figure 4(d)). The models were apparently not able to detect the gaze marker when the background was made of concrete (see Figure 4(e)). When the (red) gaze marker overlays a red element (i.e., a car), the frame was mislabeled by the models. This probably could have been prevented by choosing a suitable gaze marker that is detectable across different backgrounds. Instances where manual labelling was wrong are very diverse and mostly seem to depend on the sequence (i.e., outliers were more frequently erroneous).

### Inference

We inferred the entire dataset (396 K images) through Model v1, who had showed the best results both during in-training validation and in the additional hold-out validation. The process was carried locally on a MacBook Pro (16 inch, 2019, 2,3 GHz Intel Core i9 with 8 cores, 64 Go 2667 MHz DDR4 and an Intel UHD Graphics 630 card with 1536 Mo) and took approximately 35 hours. The output was a .csv file containing the frames’ filenames in the first column and their assigned labels in the second. A separate file was generated separately for each participant’s video.

### Data Post-Processing and Data-Visualization

**Figure 5.**
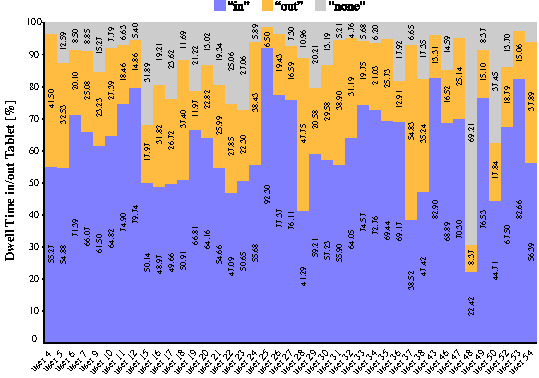
Results: Dwell time in/out of the tablet per user

After inference, we calculated dwell time for each participant on the tablet by summing up all labels and processing the .csv files using the free and open-source statistical analysis software Jamovi (see Figure 5). The average ratio of time that the gaze point was located “in” the tablet by all 41 participants was M = 61.83% (SD = 13.99). The average ratio of time that the gaze point was located “out” was M = 24.74% (SD = 10.24). On the remaining 13.43% (SD = 12.22), the gaze point could not be resolved by the MET device and the model found there to be “none” gaze marker in the frames. The individual result for each participant is shown on Figure 5.

**Figure 6.**
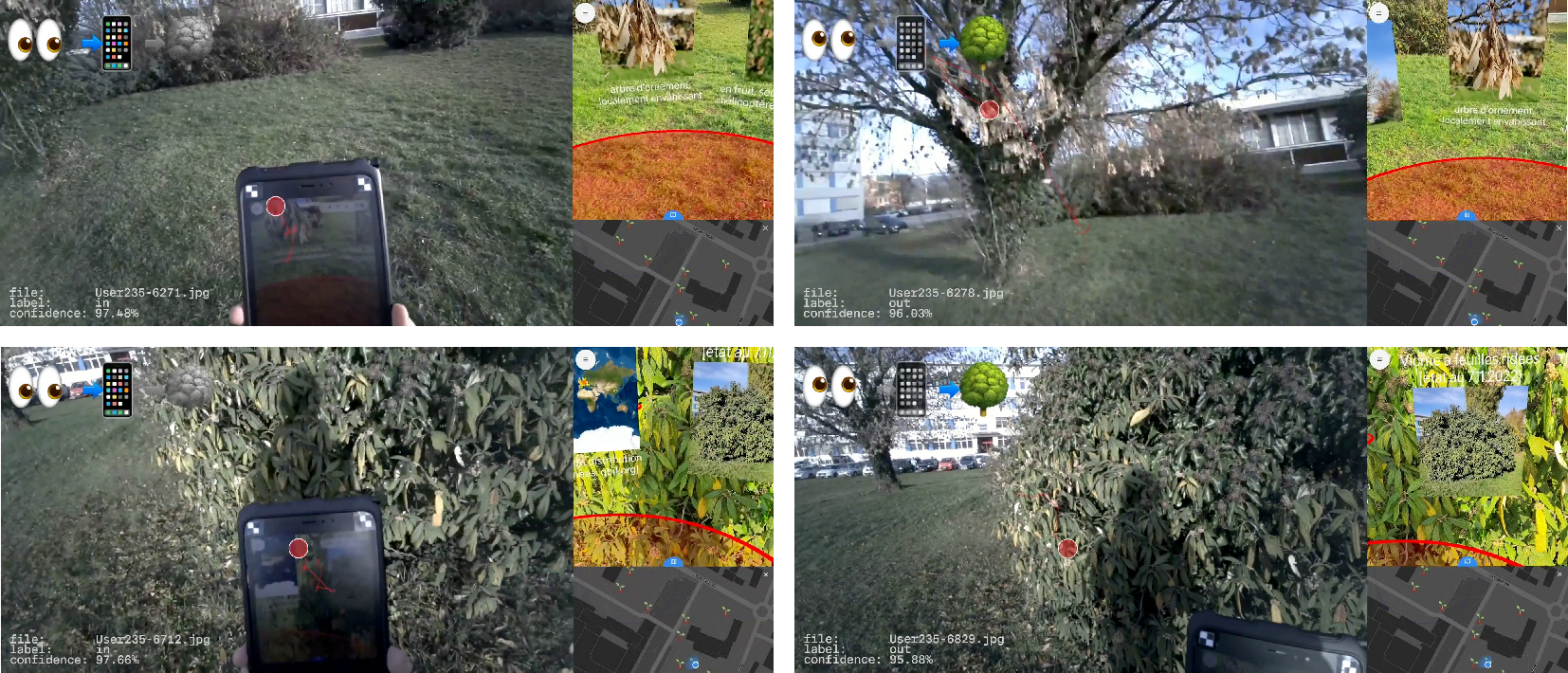
Screen captures of a post-processed video with engraved labels and tablet screen capture. The video is visible at the url: https://vimeo.com/912181285 (accessed on the 15th of March 2024).

We were further able to recreate a data visualisation video by engraving the inferred labels on each frame and assembling them back into a sequential video. We also integrated a screen capture of what is displayed on the participant’s tablet. This provides an insightful way to visualize the discovered knowledge on the source data itself (see Figure 6). A sample video can be seen at the following url: https://vimeo.com/912181285 (accessed on the 15th of March 2024).

## Results

In this study, we aimed to develop a method to measure participants’ dwell time on the tablet screen. After pre-processing our data, manually labelling a training set, fine-tuning a model by training on our data and evaluating the model with a hold-out set, we obtained a multiclass classification model that performs slightly more accurately than manual labelling (99.34% vs 99.16%). Thanks to this model, we were able to infer and label a large and noisy dataset of 396 K frames in 35 hours of autonomous computation process as opposed to the estimated 141.5 hours of manual labor it would have required otherwise. Our method enables efficient AOI dwell time analysis on MET data by automating the prohibitive and time-consuming manual data annotation process (24; 38; 41). It’s also important to consider that this work-time ratio depends on the amount of data: the more data there is to analyze, the more such an automatic process becomes relevant. Even when accounting for the time necessary to the manual labelling of the training set (2h24) as well as for the setting up of the training, the time saved is already considerable, not to mention the fact that 141 hours of such a repetitive, laborious task would have been mentally exhausting. By running our dataset through the most accurate model we trained, we were able to calculate the ratio of dwell time each of our 41 participants spent on the tablet screen, which is an important indicator in the context of our broader research on location-based augmented reality for biodiversity education, and educational technology in general. The monitoring of dwell time interaction with our system helped us gain a deeper understanding of its strengths and limitations and is an important measure that may prove useful to decision-makers in the context of education and deployment towards a younger audience.

Overall, the image classification approach we developed seemed best suited and more cost-effective than using an object detection or semantic segmentation model for the task at hand. Based on their respective SOTA performance, the approach might also be more accurate, although a dedicated comparative study would be necessary to ascertain this. Because image classification is a simpler task than object detection of semantic segmentation, the results are likely to be more accurate, outperforming even manual labelling in addition to saving time. Image classification training data is also easier to produce, since it requires only labels as opposed to the drawing of localized bounding boxes for each frame. By relying upon ViT and its inferential understanding of an AOI’s thresholds rather than object tracking algorithms, our method bypasses some of the other methods’ most common pitfalls (need for data post-processing, lack of accuracy, minimal AOI size requirement, etc.). It avoids the main disadvantages of using an object detector such as biases caused by rectangular/non-fitting and overlapping bounding boxes causing the gaze point to be miscategorized. Our method also avoids the main disadvantages of using a semantic segmentation model such as the complexity of annotating the training and test sets, additional steps for cross-referencing the data, and lesser accuracy.

Additionally, this approach could be further developed and applied to more complex tasks. This type of fine-tuned classification model could be trained to detect virtually anything that can be expressed through labels, beyond localized objects and AOI analysis. We discuss some of these perspectives in the following “discussion” section.

## Discussion

The method we describe in this article aims to address the current gap in the availability of efficient tools for the analysis of dynamic AOIs. Through this, we intend to facilitate the use of mobile eye tracking tools for naturalistic studies, which may be discouraged by this gap. By building alternative methods that help with the automatic detection and annotation of visual attention to AOIs, eye tracking will become more convenient to use for naturalistic studies. Using an image classifier–and particularly a ViT model–is an efficient way to make discoveries from egocentric video with gaze data. By dedicating a fraction of the time needed to process data manually, we show how a classification model can be used to extract the desired knowledge and perform AOI analysis on noisy MET data. The proposed method provides indexed labels for the frames of a given video, which makes it possible to perform various analyses (i.e., average glance duration, number of fixations, etc.). Glance duration and number of fixations can be calculated on the labelled data with simple scripts, but because we did not need them, we did not calculate them. This method does require time to familiarize oneself with deep learning tools and jargon, but people that are proficient with a programming language may be able to grasp it and use it for their own analysis.

As already mentioned, object detection and semantic segmentation are viable alternative to perform similar analysis and more. Compared to those, our approach, based on image classification, may pose limitations to the depth of possible analysis, but this is made up for by its relative convenience of use. Using an image classification model does require one extra step: data must feature a visible overlaid gaze marker, which can be performed within the manufacturer’s analysis tools directly. Again, the cost of this extra step of work seems balanced by the absence of need for geometric calculation to cross-reference gaze data with that of the calculated AOIs, which can be substantial, especially in the case of non-rectangular AOIs. Object detection and semantic segmentation have their own limitations such as the generation of rectangular-shaped AOIs only (in the case of object detection), or the additional nontrivial steps required (in the case of semantic segmentation), in addition to reduced SOTA accuracy. It appears that limiting the process to classifying images (rather than also localizing or segmenting the AOIs, which are additional sources of bias) may provide more accurate results.

These optimistic assessments must however be considered along with a series of limitations inherent to the approach. The approach heavily relies on data featuring rendered gaze points, which may cause problems when the marker color matches the object or the background. The rendering style of the gaze point may influence the results, and study would have to be conducted to determine best practice advice. The presented use case considers a single AOI with relatively simple geometry, which is not representative of most studies that use dynamic AOIs analysis, and the hold-out test was conducted on a single recording. A study would have to show whether the proposed method performs with more complex geometries. An approach based on attention maps would also help understand whether the model learns a geometric representation of AOIs or if it makes prediction based on the scene content, around the gaze point. Finally, it is important to consider the context of the very high level of customization of MET data. In this type of studies, data is highly specific and may present unique and unexpected traits in every situation. For this reason, while we think our approach would be transferable to other cases, this has yet to be demonstrated by applying it in other use cases, with data collected under different conditions. A methodical study would have to be conducted to weigh the costs and benefits of each approach.

While it may not be suitable for every AOI analysis situation, we think the simplicity of the approach also gives it potential to be applied to more sophisticated analysis. It shows the necessary versatility for the analysis of in-the-wild complex and dynamic scenery that other approaches may lack. In the use case presented in this study, the model could have been trained to classify more detailed features such as the species of the observed plants, or the type of content displayed on the screen. This also includes cases where the classifiable property of a situation is embodied holistically, in the entirety of the scene (e.g., some types of land use, weather, human interaction and behavior), as opposed to objects whose visible contours can be traced. The approach can be scaled-down to discern a subcategory (e.g., “blue-eyed wolf”) or scaled-up to detect a super category containing many–visually diverse–examples (e.g., “animals”). It could also be used to classify similarly looking habitat types (e.g., savanna, steppe, prairie, pampas are all grasslands, but only a trained biologist can easily distinguish them), etc. A classifier should be able to pick up any feature that’s visible to a trained human eye without being easy to describe with words or logical conditions, such as the weather (e.g., a rainy sky is generally immediately recognizable to the human eye, regardless of the multitude of formal features it contains). Arguably, this approach can be repurposed by using a variety of data. It is applicable in situations where the extraction of actionable knowledge from noisy data (not limited to MET data) would be difficult otherwise. In future research, we would like to put these prospects to the test by developing the approach to perform more demanding analysis.

In brief, the method consists in applying a known, non-specific classification pipeline to a known problem that is specific to mobile eye tracking: dynamic and nonlinear moving AOI analysis. By combining a streamlined deep learning approach to address a methodical gap in the field of MET and AOI analysis, we obtain a somewhat innovative, automated, efficient, and structured approach.

### Code Availability

All the code used by the authors is under the MIT License and it is available at the url: https://github.com/MediaComem/ViT-for-multiclass-image-classification

The trained models are under the Apache 2.0 license and are available at the URLs: https://huggingface.co/julienmercier/vit-base-patch16-224-in21k-mobile-eye tracking-dataset-v1 https://huggingface.co/julienmercier/vit-base-patch16-224-in21k-mobile-eye tracking-dataset-v2

The dataset used to train the models is under a CC-BY-NC-ND-4.0 license and is available at the url: https://huggingface.co/datasets/julienmercier/mobile-eye tracking-dataset-v2

### Ethics and Conflict of Interest

The author(s) declare(s) that the contents of the article are in agreement with the ethics described in http://biblio.unibe.ch/portale/elibrary/BOP/jemr/ethics.html and that there is no conflict of interest regarding the publication of this paper.

### Acknowledgements

This research was supported by the Swiss National Science Foundation: NRP 77 “Digital Transformation” (project number 407740_187313).

We wish to thank Trevor Lynn of Roboflow for his advice on ViT, Iñaki Gomez Mowatt for his help during implementation, Yoann Douillet of the Media Engineering Institute for his help with mobile eye tracking data collection, and Prof. Giovanni Colavizza for providing ideas in this article. ­­

## Figures and Tables

**Figure:**
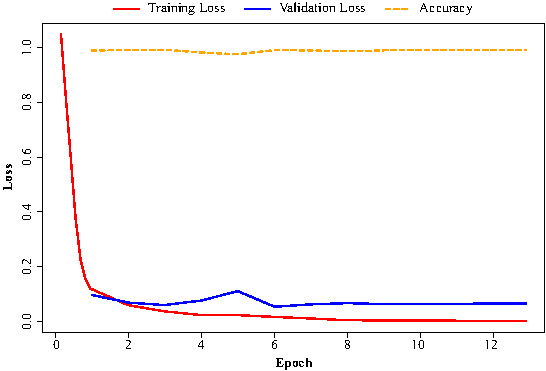
The accuracy curve (dotted orange on the graph) reached 98.85% at the end of epoch no. 1 and almost capped from thereon. Peak accuracy was reached after epoch no. 3. The validation lost lowest scores were also observed at epoch no. 3 (for the first training session) and epoch no. 6 (for the second).

## Appendix A

### Image Data Embedding in Deep Learning

The performance of deep learning architectures notably comes from their ability to process large data (both in the amount of data points or of samples) and to create meaningful representations–also known as embeddings–of high-dimensional data (in the number of variables or features for each data point). High-dimensional data can be intuited as “extremely high-resolution data”, and has surprising properties (Aggarwal et al., 2002). Visual data follows a typical structure that features spatial and temporal coherence (Khan et al., 2022). Consider two pictures that each represent a different human. In a high-dimensional space, the pictures are likely to share some specific, meaningful distance metrics (sometimes referred to as hidden topics or internal representations) along some dimensions, whereas a picture of a wolf will be more distant (as can be visualized on a t-SNE graph). However, if the wolf and one of the humans’ pictures both have blue eyes, their data points will share specific meaningful distance metrics along some dimensions that the two humans’ data points will not. However, processing high-dimensional data, let alone in large amounts, has prohibitive computational cost. Therefore, a critical aspect is dimensionality reduction, which involves the transformation of data from a high-dimensional space into a low-dimensional space, in a way that the reduced data retains some meaningful (latent) properties. The key is to identify which dimensions are most useful given a specific task (i.e., similarities between pictures of humans or blue-eyed mammals?). Various architectures adopt various strategies for data embedding. In the following, we provide general intuitions on CNN and Transformer architectures to highlight their key differences.

## Appendix B

### Convolutional Neural Network

Convolutional Neural Networks (CNNs) perform convolutions to compute the similarity between two signals. It can be pictured as the application of filters over portions of an image to detect features. The convolutional layers encode high-level features (e.g., humans pictured under varying conditions) into vector embeddings, which are numerical representations of data that retain their most meaningful features. These embeddings allow data points featuring semantically similar things (e.g., pictures of humans, pictures of blue-eyed mammals) to be clustered in a high-dimensional space, which is a non-trivial task in the raw, full pixel space. CNN’s drawback is its computational power needs. Convolutions treat all pixels of input images equally, regardless of their importance. It’s also generally little aware of context and struggles to relate concepts that are spatially distant in an image (Wu et al., 2020).

## Appendix C

### Vision Transformer

The performance, flexibility and customization of deep learning algorithms has been continuously improving over the past few years. Introduced in 2017, the Transformer architecture (Vaswani et al., 2017) has gained world recognition through the popularization of large language models such as GPT-4 and LLaMa. Developed in 2020, Vision Transformer or ViT (Dosovitskiy et al., 2021) is a variation for computer vision tasks. ViT is based on the use of self-attention mechanisms instead of CNNs’ convolutions to build data representation. Self-attention works with sequential data and allows the representation of the interaction of each item of a sequence with all other items. To become sequential, high-dimensional data is reduced by splitting the image in non-overlapping patches (of 16 by 16 pixels each, in the implementation we used) that are mapped and transformed into vector embeddings by the self-attention layers. These layers update each item of the sequence by retrieving contextual information from the complete sequence, which produces a deep semantic representation of each data point. The data can be described as points situated in a multidimensional space, where similar data (i.e. pictures of humans) is clustered (Khan et al., 2022). The main difference between CNN and ViT is that self-attention layers are dynamic filters (constantly recalculated) whereas convolutional layers are static filters. ViT represents images as semantic visual tokens can model relationships between tokens that are spatially distant. It allows to weigh different image parts based on their contextual importance (Wu et al., 2020). The choice of using ViT or CNN relies on many factors, including the task, available data, computational power needs, and training time. A literature review found ViT to be lighter, consuming fewer computational resources and taking less training time than CNN (Maurício et al., 2023). ViT is found to be particularly performant when pretrained with self-supervision on large unlabeled data, then fine-tuned to a specific task. Fine-tuning builds on knowledge that an existing model has learned from previous data. Fine-tuning a pretrained ViT model with a small labeled dataset is reportedly more efficient and accurate (Dosovitskiy et al., 2021), and to our knowledge has not been applied to MET data analysis yet. Most recent research combines benefits of both CNN and ViT by using convolutions to create feature maps fed as patches in ViT rather than raw images.

## Appendix D

### Training results.

**Table:**

**Epoch**	**Step**	**Training Loss**	**Validation Loss**	**Accuracy**
1	73	0.1179	0.0977	98.85%
2	147	0.06	0.0693	98.98%
3	219	0.0376	0.0604	99.11% ^1^
4	292	0.024	0.0769	98.09%
5	366	0.0236	0.1111	97.45%
6	440	0.0172	0.0542	98.98% ^2^
7	514	0.0114	0.0630	98.85%
8	587	0.0051	0.0674	98.60%
9	661	0.0044	0.0640	98.85%
10	735	0.0037	0.0646	98.85%
11	809	0.0034	0.0652	98.85%
12	882	0.0032	0.0656	98.85%
13	949	0.0032	0.0657	98.85%

^1^ After epoch 3, training reached best accuracy overall (as evaluated on a test set that was split from the training set) and the checkpoint was saved as Model v1. ^2^ After epoch no. 6, training reached best accuracy of the second training run and the checkpoint was saved as Model v2.
